# Combinatorial Activation and Repression by Seven Transcription Factors Specify *Drosophila* Odorant Receptor Expression

**DOI:** 10.1371/journal.pbio.1001280

**Published:** 2012-03-13

**Authors:** Shadi Jafari, Liza Alkhori, Alexander Schleiffer, Anna Brochtrup, Thomas Hummel, Mattias Alenius

**Affiliations:** 1 Department of Clinical and Experimental Medicine, Linköping University, Linköping, Sweden; 2 Research Institute of Molecular Pathology (IMP), Vienna, Austria; 3 Department of Neurobiology, University of Vienna, Vienna, Austria; The Rockefeller University, United States of America

## Abstract

The mechanism that specifies olfactory sensory neurons to express only one odorant receptor (OR) from a large repertoire is critical for odor discrimination but poorly understood. Here, we describe the first comprehensive analysis of OR expression regulation in *Drosophila*. A systematic, RNAi-mediated knock down of most of the predicted transcription factors identified an essential function of acj6, E93, Fer1, onecut, sim, xbp1, and zf30c in the regulation of more than 30 ORs. These regulatory factors are differentially expressed in antennal sensory neuron classes and specifically required for the adult expression of ORs. A systematic analysis reveals not only that combinations of these seven factors are necessary for receptor gene expression but also a prominent role for transcriptional repression in preventing ectopic receptor expression. Such regulation is supported by bioinformatics and OR promoter analyses, which uncovered a common promoter structure with distal repressive and proximal activating regions. Thus, our data provide insight into how combinatorial activation and repression can allow a small number of transcription factors to specify a large repertoire of neuron classes in the olfactory system.

## Abstract 2

A systematic analysis reveals a regulatory network controlling selective odorant receptor expression and neuronal diversity in *Drosophila*.

## Author Summary

Our nervous system has a daunting diversity; it contains 100 billion neurons that all have defined functions and connections. To address how neuronal diversity is produced, we have turned to a complex but defined set of neurons, the *Drosophila* olfactory system located in the antenna. This system contains 34 neuron classes with different functions and connections, each defined by the unique odorant receptor they express. We set out to identify the transcription factors (regulatory genes) that are required for each class to express the correct odorant receptor. We find that seven transcription factors are continuously required in different combinations for the expression of all tested 32 odorant receptors. We also show that these transcription factors can both turn on and turn off odorant receptor genes, making the expression regulation more economical. We conclude that dual use of a small set of factors, which are always on in the neuron, can define its functional class and thereby produce diversity in the nervous system.

## Introduction

The external world is perceived by peripheral neurons that each expresses only one or a stereotyped set of receptors from a large genomic repertoire [1]–[4]. The selective receptor expression ensures the specific function of each sensory neuron and produces a daunting diversity of sensory neuron classes. However, little is known about how the neuron class-specific receptor expression is controlled.

In the mouse olfactory system, each olfactory sensory neuron (OSN) chooses to express one odorant receptor (OR) out of approximately 1,200 OR genes [5]. OR choice in mammalians is in part a stochastic process restricted by the developmental context, which is manifested as restricted zonal expression patterns of each OR [6]. The zonal patterns can be resembled by the expression of transgenic OR promoters [6],[7] and raises the possibility that there are transcription factors (TFs) that in combinations or in gradients specify mouse OR expression. Two TFs, Lhx2 and Emx2, have been identified as general regulators of OR expression [8]–[10], but the identities of the TFs that regulate specific mouse ORs are unknown, because the large size of the OR repertoire makes systematic analysis of TF phenotypes cumbersome and specific defects difficult to detect.

In similarity to mammals the *Drosophila* ORs are expressed in a salt and pepper pattern within domains of the antenna OSNs (Figure S3) [11]. *Drosophila* OR expression create 34 OSN classes with a stereotype neuronal number and location [12]–[14], suggesting a strictly predetermined process. The large number of OSN classes and precise OR regulation makes the *Drosophila* antenna an extraordinary system to study how ORs are regulated and how a large number of neuron classes are specified. To date, only two TFs, Acj6 and Pdm3, has been shown to specify a subset of *Drosophila* ORs [15],[16]. However, no systematic approach has yet been undertaken to address the regulatory mechanism of OR expression.

To address how the olfactory system specifies the unique OR identity of a large number of sensory neurons we have performed the first systematic genetic (directed RNAi) screen for direct regulators of *Drosophila* OR expression. Hereby, we have identified a set of only seven TFs that regulate the complete OR collection of the adult *Drosophila* olfactory system. We provide a systematic analysis to demonstrate how these TFs employ multiple strategies to specify OR class identity.

## Results

### A Systematic RNAi Screen Identifies Seven TFs That Regulate OR Expression

**Figure 1:**
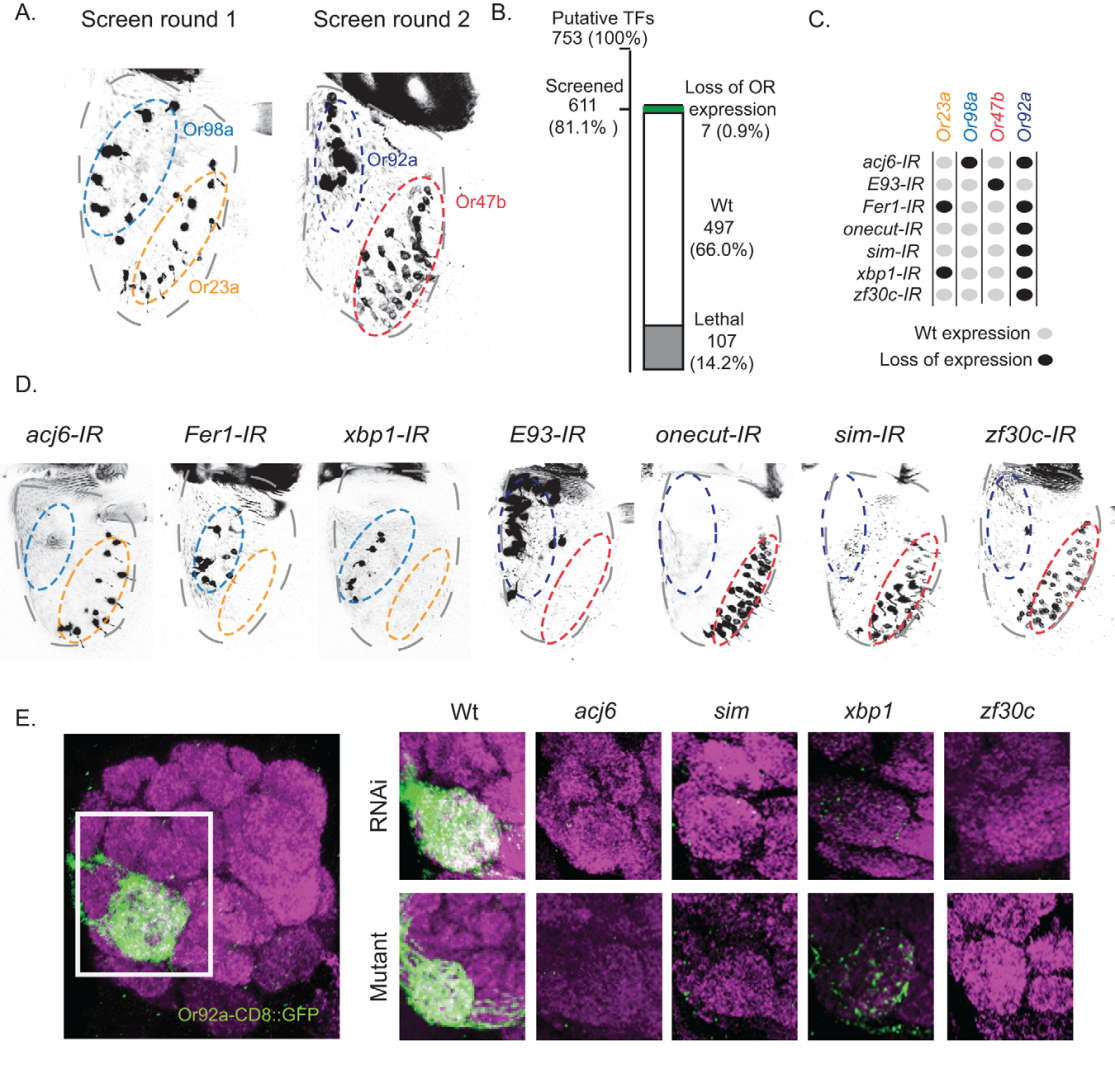
An RNAi screen identifies seven TFs required for OR expression. (A) Whole mount preparations of antenna from the two screening rounds (GFP in black). In the first round, expression of *Or98a-CD8::GFP* and *Or23a-CD8::GFP* in two mid-antennal domains (light blue and orange oval) were analyzed. In the second round, *Or92a-CD8::GFP* expression in the most proximal (dark blue oval) antenna domain and *Or47b-CD8::GFP* expression in the most distal (red oval) antenna domain were analyzed. (B) Statistics from the screen is depicted as a graph, summarizing the number of IR lines that did not affect OR expression (Wt, white), led to lethality (Lethal, grey) or lost OR expression (Loss of OR expression, Green). (C) Phenotype summary for the seven TF-IRs and the analyzed OSN classes, wild-type OR expression (grey dots) and loss of OR expression (black dots). (D) Antenna from each TF-IR with representative OR expression phenotypes. (E) Whole mount antennal lobe with the Or92a-CD8::GFP OSN projections shown in green and the synaptic marker, nc82, delineating the glomeruli of the antennal lobe, in magenta. The boxed region indicates the antennal lobe area in the right panel, which compares the RNAi and mutant phenotypes of *acj6*, *sim*, *xbp1*, *zf30c*. Note the loss of *Or92a* in both the mutant and RNAi lines.

In mammals and insects, the majority of OSNs each express a single OR gene out of a large genomic repertoire. To identify the TFs that are necessary for proper OR expression in *Drosophila* we used the transgenic UAS-driven inverted repeats (IRs) from the Vienna *Drosophila* RNAi Center (VDRC) [17] to interfere with the 753 annotated putative TFs in *Drosophila* (www.flytf.org) [18]. The TF-IRs were expressed in postmitotic OSNs by *pebbled*-Gal4 [19], and OR expression was visualized by direct OR promoter fusions with *CD8::GFP* (Figure 1A). In two separate rounds we analyzed the RNAi effect on the expression of four representative OR classes: *Or92a* and *Or98a* for basiconic OSNs in the distal and central antennal region, Or23a and *Or47b* for trichoid OSNs in overlapping proximal antennal domains (Figure 1A). We found 611, 81.1% of the TFs, to be available as RNAi lines in the VDRC library and expression of which lead to lethality of another 14.2% (Figure 1B). Of the remaining 504 gene knock downs (TF-IRs), we identified seven that resulted in a strong and highly penetrant loss of OR expression: *acj6-*, *E93 (Eip93f)-*, *Fer1-*, *onecut-*, *sim-*, *xbp1-*, and *zf30-IRs* (Figure 1C and 1D).

**Figure 2:**
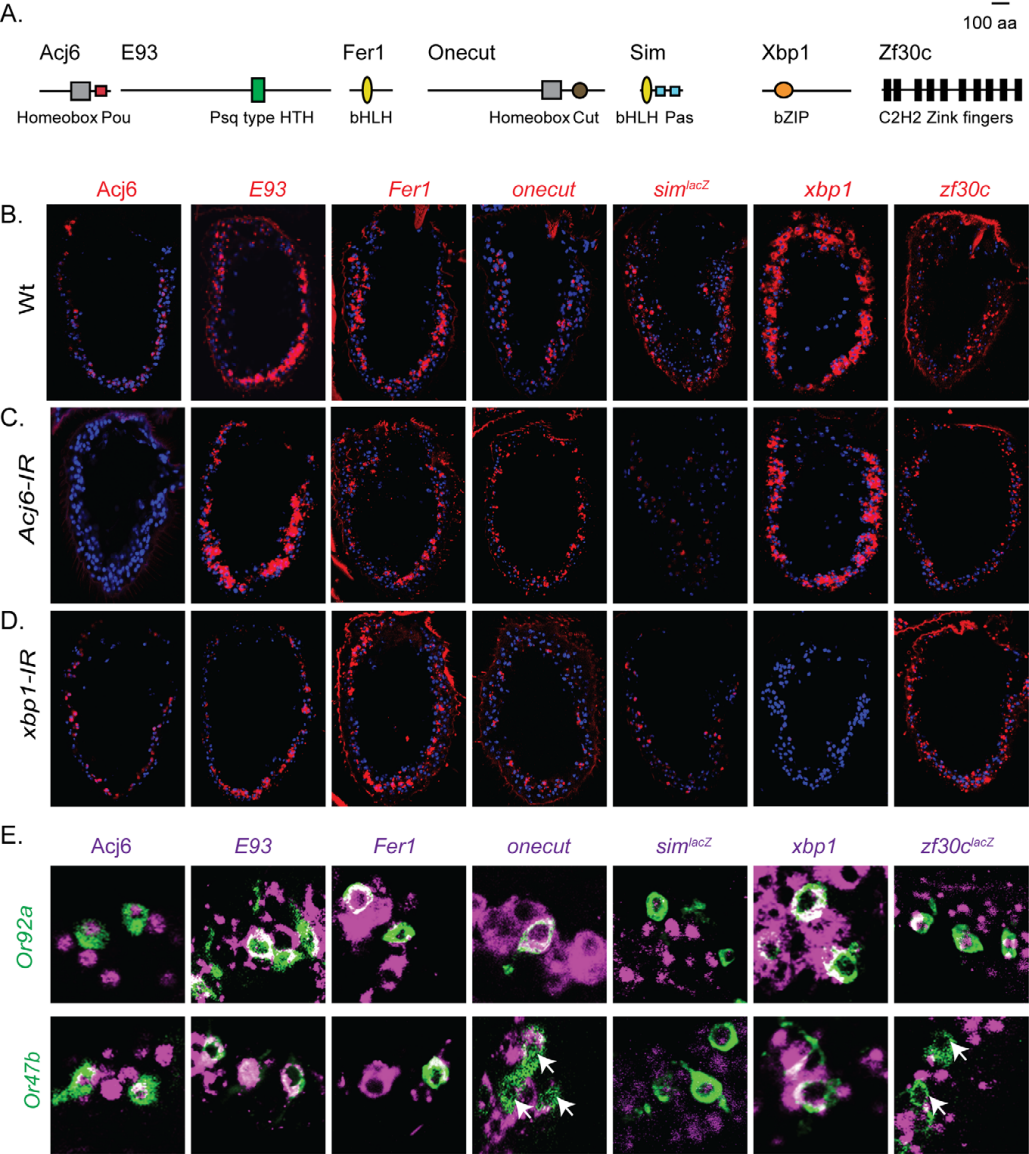
Expression of OR gene regulators in the adult *Drosophila* antenna. (A) The identified TFs belong to different protein families as indicated by their protein domain organization. (B) In situ hybridizations and immunohistology on wild-type antenna sections showing the expression pattern of each TF (red) counterstained with the nuclear marker DAPI (blue). (C,D) RNAi-mediated reduction of Acj6 (C) and Xbp1(D) does not affect the overall expression pattern of the other six TFs. (E) Expression of the TFs (magenta) in either *Or47b-CD8::GFP* or *Or92a-CD8::GFP* (green) expressing OSNs. Note, that the Or47b expressing OSNs lack expression of onecut and zf30c (arrows).

To exclude false positives caused by off-targeting and insertion mutagenesis, multiple IR lines from VDRC (http://stockcenter.vdrc.at), National Institute of Genetics (NIG-Fly, http://www.shigen.nig.ac.jp/fly/nigfly), and Transgenic RNAi Project (TriP, http://www.flyrnai.org) that corresponded to each of the seven genes were analyzed. All constructs gave rise to identical phenotypes (Table S1), supporting the specific knock down of each TF. In addition, mutant analysis for the TFs with available defined mutant alleles (*acj6^6^*, *xbp1^k13803^*, *sim^H9^*, and *zf30c^k02506^*) gave similar phenotypes compared to the RNAi lines (Figure 1E; Table S1). Finally, direct expression analysis on each IR background demonstrated knock down of the corresponding TF (Figures 2 and S1). These results indicate that these seven TFs are critical regulators of OR expression.

### Overlapping Expression of the Seven TFs

All identified TFs belonged to different protein families (Figure 2A): Acj6 (POU-Homeobox; Hox), E93 (Psq like helix-turn-helix; HTH), Fer1 (basic helix loop helix; bHLH), Onecut (cut-Hox), Sim (PAS-bHLH), Xbp1 (bZIP), and Zf30c (C2H2; zinc finger). Two of the genes, *zf30c* (*zinc finger at 30c*), which encodes a protein with ten C2H2 zinc finger domains and *Fer1* (*Forty eight related 1*), encoding a bHLH factor, had not previously been characterized.

We next asked whether expression of the seven TFs correlated to the OR expression domains or sensilla groups. There are three main groups of sensilla, basiconic, coeloconic and trichoid, differently distributed across the antenna [20]. In situ hybridization and immunohistochemistry demonstrated that all seven TFs were expressed in the adult antenna in various patterns (Figure 2B). Each pattern showed little restriction to domains or sensilla groups and none of the TFs were expressed in only one or only a few OSN classes (Figure 2B). Two of the TFs, *acj6* and *xbp1*, were ubiquitously expressed and might regulate OR expression more indirectly via any of the other five TFs. When analyzed, no obvious differences in strength or distribution of *E93*, *Fer1*, *onecut*, *sim*, and *zf30c* expression were found in the *acj6*- and *xbp1*-IRs, indicating that the seven TFs might be directly required for OR expression (Figure 2C and 2D).

To address the extent of coexpression between the seven TFs, we analyzed each TFs expression in two OSN classes (Figure 2E). In *Or92a* OSNs, all seven TFs were expressed including *E93*, the TF that was not required for *Or92a* expression. Similarly, Or47b neurons expressed *E93*, the only TF required for expression, and *acj6*, *Fer1*, *sim*, and *xbp1*. These data show that the seven TFs are expressed in broad and overlapping populations of mature sensory neurons, which do not correlate with sensilla groups or OSN classes. The lack of anatomic correlation of the expression patterns suggests that these TFs are part of a distinct regulatory network separate from the general process of antenna and neuron specification.

### The Identified OR Regulators Are Required in Adult Flies

**Figure 3:**
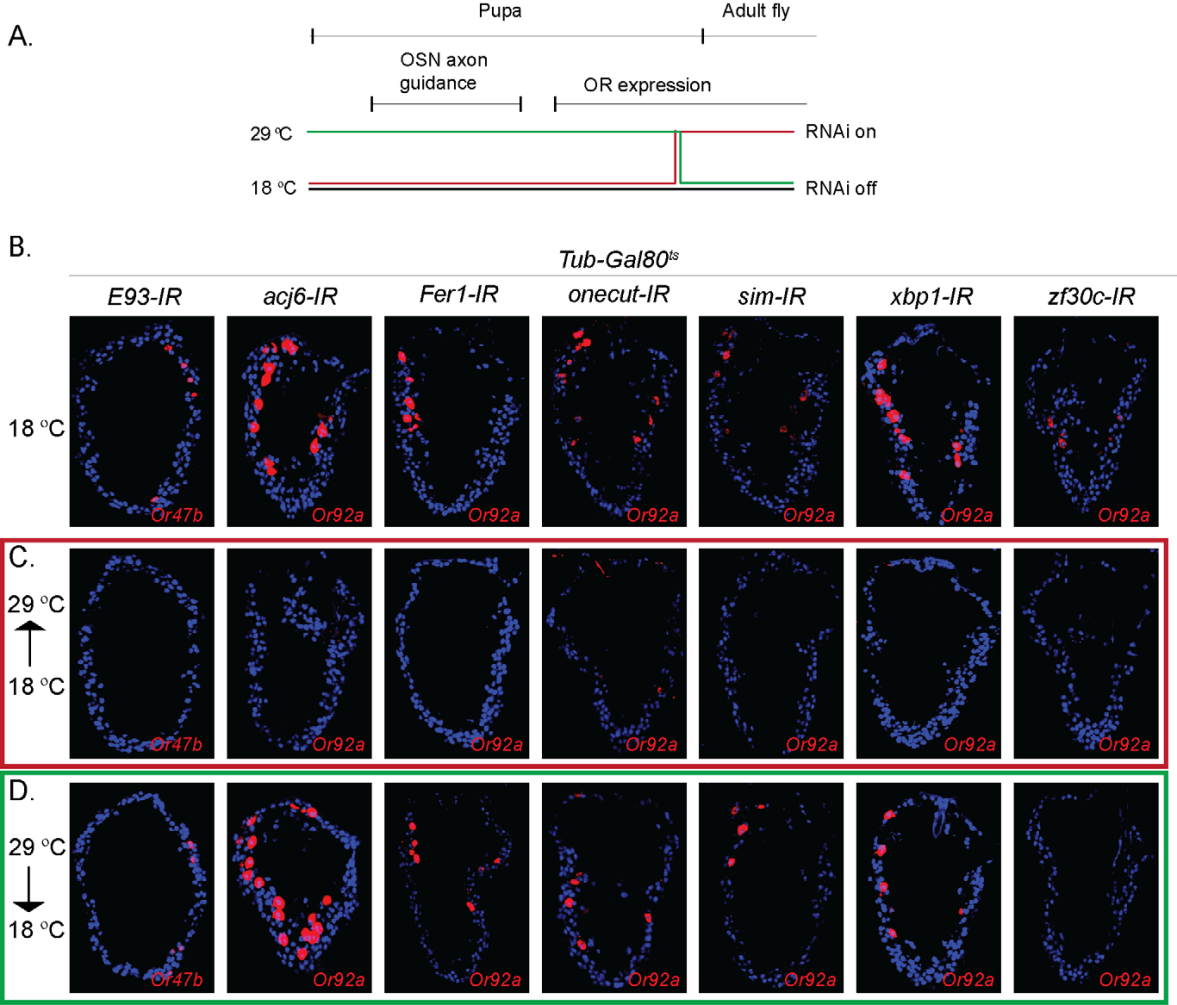
All seven TFs are continuously required for OR expression. (A) Schematic of the TARGET experiments. Flies were shifted at late pupal stage from 18°C to 29°C (red line), or from 29°C to 18°C (green line); the RNAi was induced specifically at 29°C. (B–D) *Or92a* and *Or47b* in situ hybridizations (red) counterstained with DAPI (blue). (B) With the suppression of RNAi at 18°C, the OR was expressed in all genotypes (red staining). (C) The TF knock down at the end of pupal development (shift from 18°C to 29°C) fully suppresses OR expression. (D) Developmental TF knock down (shift from 29°C to 18°C) does not affect OR expression except for *zf30c-IR*.

The onset of OR expression takes place during the second half of pupal development, after OSN axon guidance, and is one of the final steps of sensory neuron differentiation (Figure 3A). To rule out a role of the seven TFs in early OSN specification and differentiation, which could affect OR gene expression more indirectly, the pan neuronal markers, Elav and Neuroglian, were analyzed. The overall number of OSNs and axonal projections from the antenna to the brain was not affected in any of the RNAi knock downs, indicating no gross changes in sensory neuron development (Figure S2).

Next, to determine the temporal window of TF function in OR expression we used the TARGET system [21]. Here, the IR-mediated gene knock-down can be regulated via a temperature-sensitive Gal4 repressor (GAL80^ts^) (Figure 3A). At the restrictive temperature (29°C), GAL80^ts^ is inactivated, permitting Gal4 to express the *TF-IR* in all OSNs (Figure 3A). Flies maintained continuously at 18°C (no *TF-IR* expression) expressed *Or92a* and *Or47b* at the correct antennal location (Figure 3B). In contrast, when the *TF-IR* flies were shifted after the onset of OR expression to 29°C, Or92a or Or47b expression was lost (Figure 3C).

In a reverse approach, knock down of the TFs during pupal development and a reversal of the wild-type TF expression in early adult stages allowed us to distinguish between earlier developmental roles and a later function in OR gene regulation (Figure 3D). Developmental suppression of *acj6*, *E93*, *Fer1*, *sim*, *onecut*, and *xbp1* did not affect adult OR expression (Figure 3D), whereas knock down of Zf30c during pupal development reduced OR expression. These data support a view of sensory neuron development where these seven TFs possess a specific OR regulatory function and with Zf30c having an additional earlier role in OSN class specification.

### A TF Regulatory Matrix for *Drosophila* OR Expression

**Figure 4:**
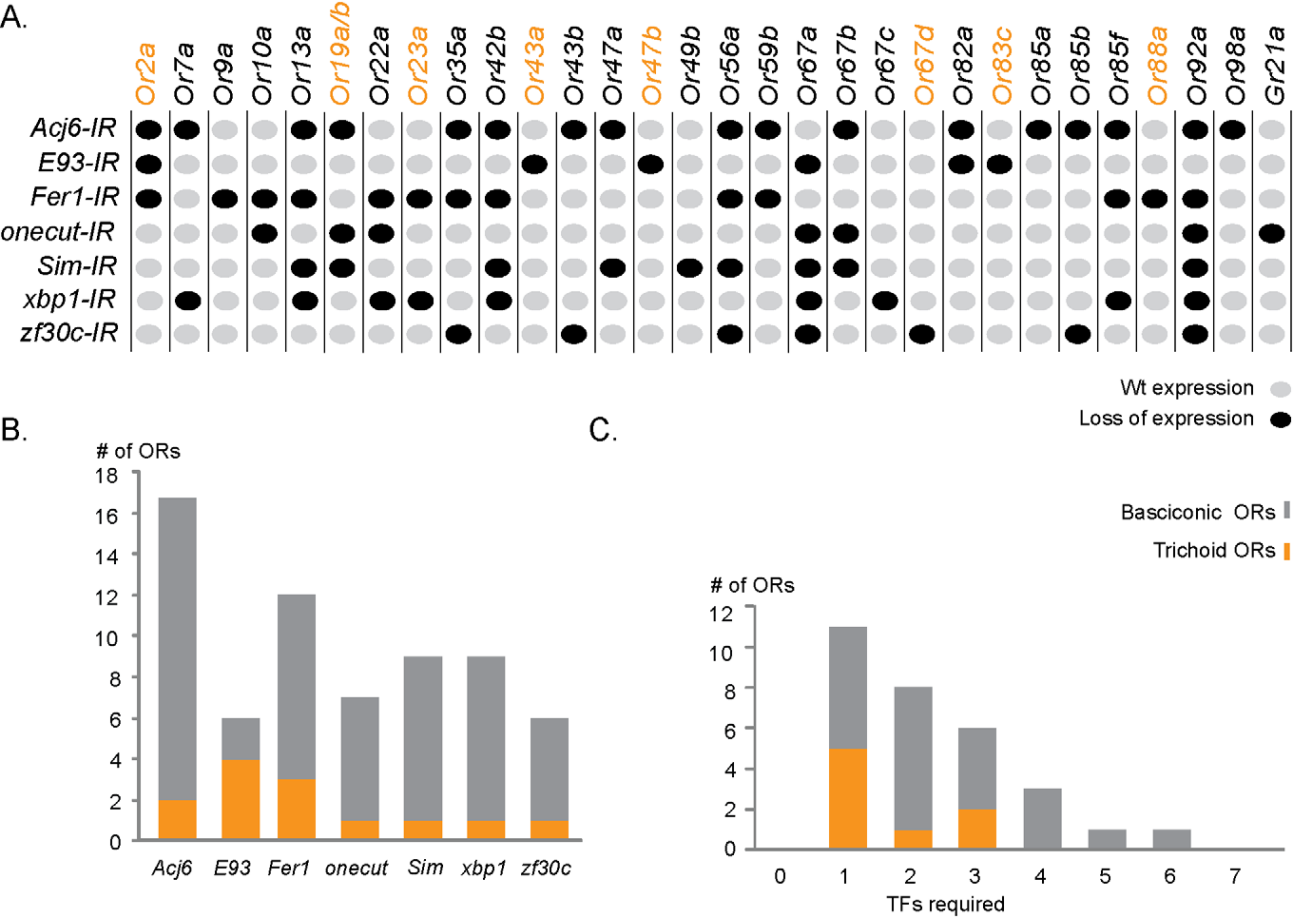
A regulatory matrix for *Drosophila* OR expression. (A) The regulatory matrix represents in situ hybridizations for 32 ORs/TF-IR, indicated as wild-type levels (gray dots) and lost (black dots) OR expression. Trichoid ORs marked in orange. (B) Bar diagram, representing number of ORs that required each TF for expression. Trichoid ORs marked as orange insets in each bar. (C) Number of ORs regulated by 0–7 TFs depicted as bar graphs. (See Table S2 for statistics and see Figure S3 for domain and sensilla arranged matrix).

To determine whether this small set of TFs can regulate the full collection of OR genes, we extended our RNAi analysis to the majority of the sensory neurons classes in the *Drosophila* olfactory system. The resulting OR expression phenotypes were assembled into a regulatory matrix (Figure 4A; for statistics see Table S2). The matrix exposed several general regulatory features. First, all 32 ORs required at least one of the seven TFs for correct expression, demonstrating a prominent role in OR gene regulation for the TFs (Figure 4A and 4C). Second, in line with the wide TF expression patterns across the antenna (Figure 2B), the TFs were required for OR expression in OSN classes indiscriminate of sensilla group or antenna location (Figures 4B and S3), supporting that the TFs disconnect the OR expression from the early antenna patterning and development. Third, the identified seven TFs were required for expression of partly overlapping sets of OR genes (Figure 4A), suggesting a combinatorial mode of OR gene regulation. Last, unique TF combinations were associated with 17 of the 32 ORs expression and each combination ranged from one to six TFs and only two additional TFs would be sufficient to resolve the remaining redundancies. Taken together these data show that the identified small set of TFs in different combinations are required for OR expression in each OSN class.

### The Seven TFs Bind to Different Combinations of Motifs Upstream OR Genes

To address, whether any of the identified TFs bind directly to the regions upstream of each OR, we exploited the well-established vertebrate DNA binding motifs of Acj6, Onecut, and Xbp1. It has been shown that *Drosophila* Acj6 and Onecut share binding properties with their vertebrate orthologs (Figure S4) [16],[22],[23]. Most vertebrate Xbp1 DNA motifs contain a 6-bp core sequence C/TCACGT [24],[25]. In mobility shift assays, recombinant *Drosophila* Xbp1 bound this core sequence (Figure S4A), demonstrating shared binding properties between the Xbp1 orthologs.

**Figure 5:**
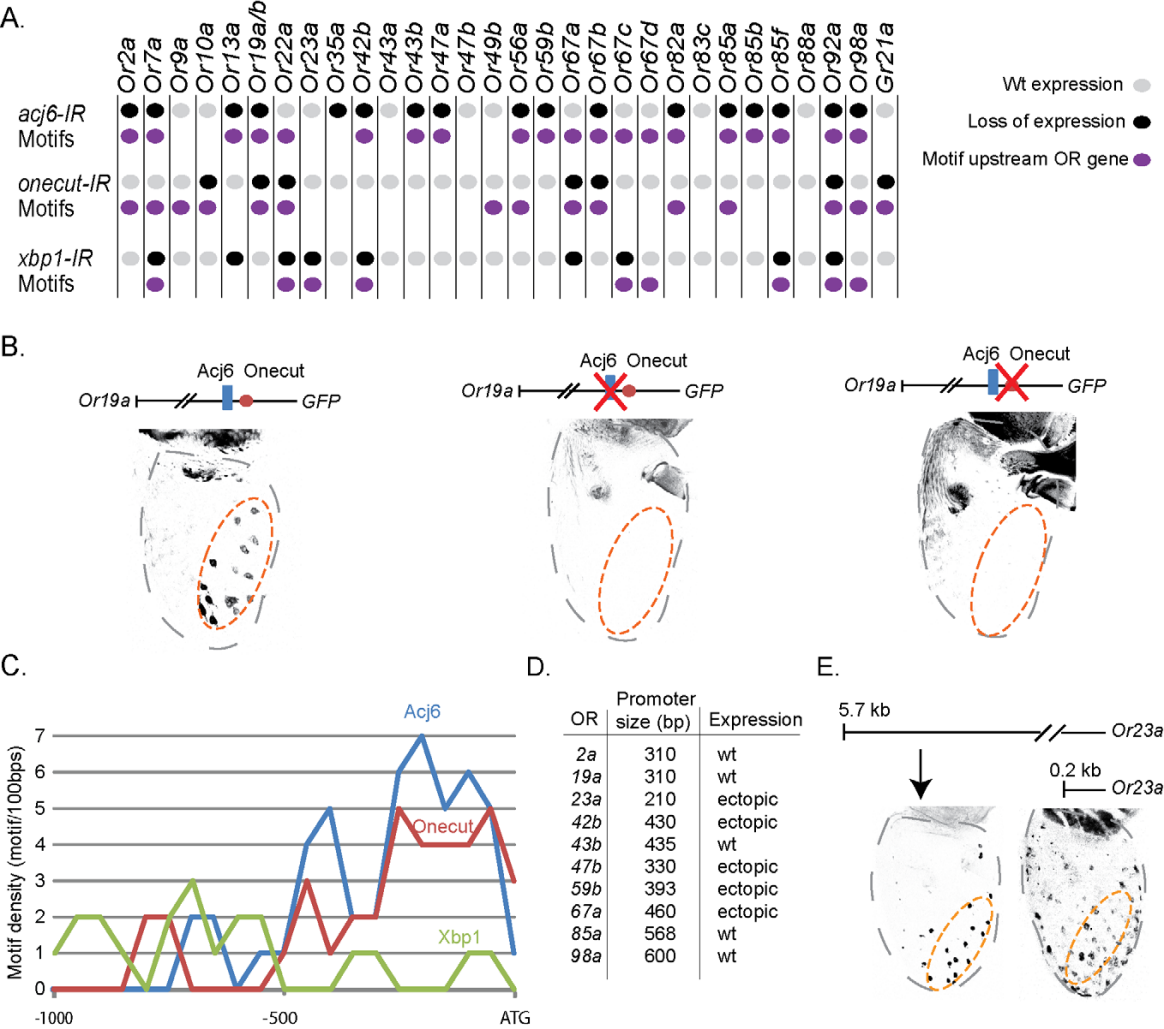
The identified TFs have binding motifs upstream the regulated OR genes. (A) Regulatory matrix for Acj6, Onecut, and Xbp1 across 32 ORs comparing RNAi phenotypes (grey and black dots) and predicted TF binding motifs upstream each OR (purple dots). (B) *Or19a* promoter construct driving CD8::GFP (black) in the correct OSN class, which is lost when either the Acj6 or Onecut motif has been mutated (C) Motif density plot across the first 1,000 bps upstream of each OR gene for Acj6, Onecut, and Xbp1 motifs. (D) Table summarizing the expression produced by the short OR promoter constructs. (E) A 5.7-kb fragment upstream of *Or23a* produces an endogenous expression pattern, whereas a short 0.21-kb fragment produces ectopic expression in a large number of OSNs across the antenna.

The Acj6-, Onecut-, and Xbp1-DNA binding motifs were used to search 1 kb upstream of 32 OR genes and identified various combinations of the TF binding motifs upstream of each OR (Figure 5A; for location of each motif see Table S3). Most OR promoter regions contained at least one binding site for the TFs required for expression (Figure 5A). The fact that some OR promoter regions lacked predicted binding sites for the required TF suggest either that the *Drosophila* TF and the vertebrate ortholog have slightly different DNA binding requirements or that the TF in these cases indirectly regulate the OR gene. In vitro binding assays for four of the OR genes showed that all motifs were recognized by the matching TFs (Figure S4C). These data together with the strong correlation between motif and OR gene activation suggest that each OR promoter is bound and regulated by different combinations of these TFs.

To address whether the motifs were necessary in vivo for OR expression, we focused on the shortest promoter region sufficient for OR expression, *Or19a* (Figure 5D). The sufficient promoter region contains both an Acj6 and Onecut motif, both TFs required for *Or19a* expression (Figure 5A). When either of the two motifs was mutated, the expression of the *Or19a* construct was abolished (Figure 5B). These results demonstrated that the motifs were necessary for promoter function and that the TFs directly regulate OR expression.

### Long Range Repression Modulates OR Expression

For Acj6 and Onecut a peak of binding motifs was observed directly upstream of the OR genes (Figure 5C), (for individual predictions see Table S3), which corresponded to a region of high sequence conservation found upstream of most OR genes (unpublished data) [26]. Transgenic constructs containing these conserved regions produced antenna OSN expression for ten tested OR promoters (Figure 5D), suggesting that a short region directly upstream of each OR gene was sufficient for expression. However, half of the short promoter constructs produced misexpression (Figure 5D and 5E); the lack of OSN class specificity implies that distal regulatory regions are required to repress OR expression in some OSN classes. The similarities in behavior for the various OR promoter constructs suggest a common OR promoter organization with a proximal region that produces expression and a distal repressive region that restricts the OR expression to one single OSN class.

### The Location of the DNA Binding Motif Determines TF Function

**Figure 6:**
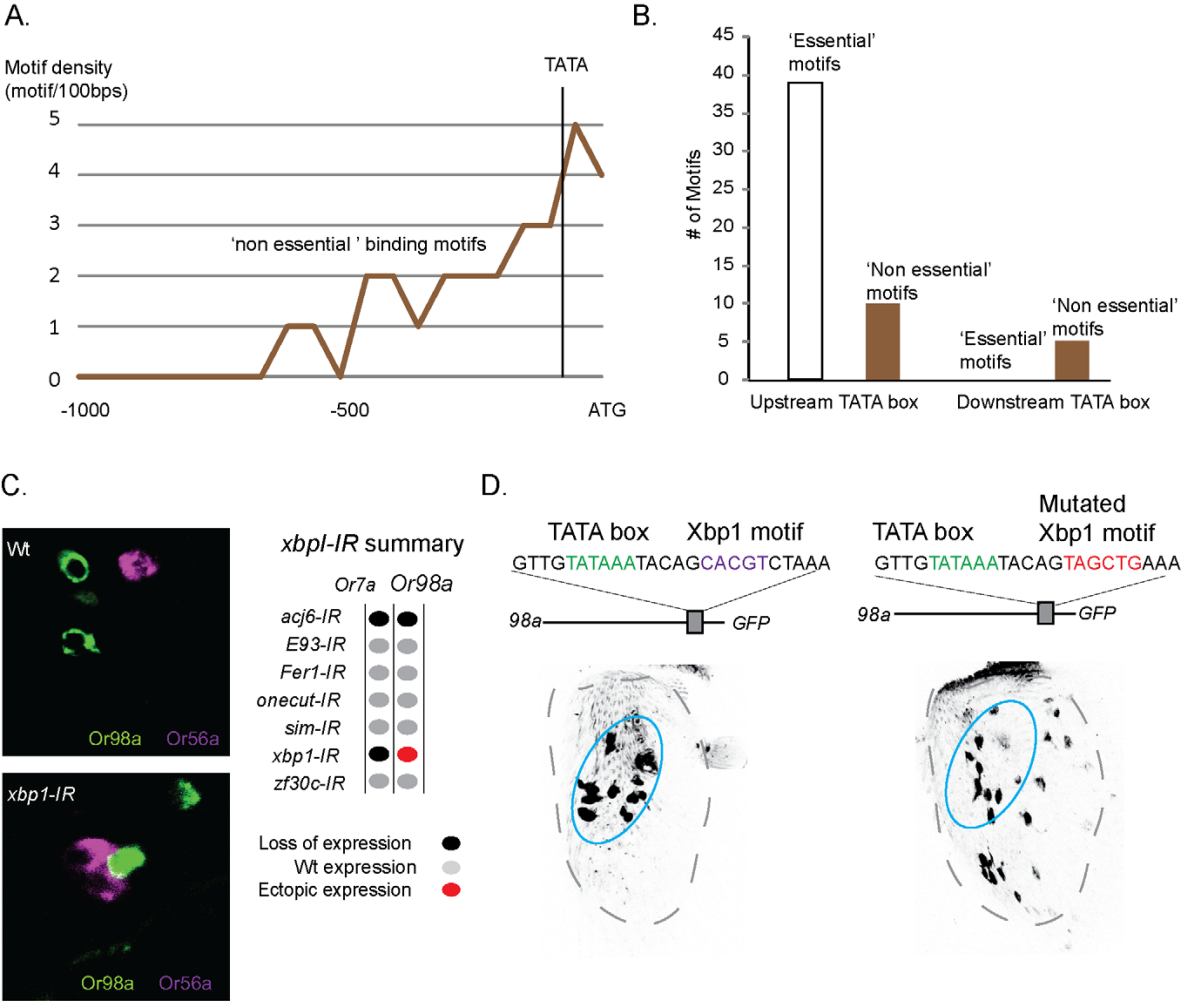
The location of the binding site upstream of the OR dictates Xbp1 function. (A) Motif density plot, showing motifs found upstream of OR genes that did not require the matching TF (see Table S3 for statistics). (B) Bar graph depicting the total number of motifs located upstream or downstream the TATA box for ORs that either require the TF (“essential”) or not (“nonessential”) for expression. (C) Double in situ labeling of *Or98a* and *Or56a* in wild type (Wt) and *xbp1-IR* antennae revealed ectopic *Or98a* expression next to *Or56a*. The RNAi phenotypes are summarized as a matrix (grey, wild-type expression; red, ectopic; and black, loss of expression). (D) One Xbp1 motif (purple) was found next to the TATA box (green) of *Or98a*. The *Or98a* promoter construct produced expression in a single domain (light blue oval, black expression). Whereas, the same *Or98a* promoter construct with a mutated Xbp1 motif (red) produced a distal expansion of the expression.

The bioinformatic analysis uncovered DNA motifs in OR promoters that did not require the matching TF for expression (Figure 5A). When the upstream locations of these “nonessential motifs” were plotted, a peak was found downstream of the TATA boxes (Figure 6A; see Table S3 for location of each motif). Conversely, all motifs upstream of ORs that required the matching TF were located upstream of each TATA box (Figure 6B), suggesting that motif location might reflect different TF functions. For example, *Or98a*, which did not require *xbp1* for expression, had an Xbp1 motif downstream of the TATA box (Figure 6D). Moreover, in *xbp1-IR* flies, *Or98a* showed ectopic expression in OSNs that normally express *Or7a* and pairs with *Or56a* (Figure 6C). The repression of Or98a and the activation of Or7a expression in the same OSN class show that Xbp1 has a dual function in the specification of OR gene expression. One simple explanation might be that Xbp1, when bound far upstream, activates expression of Or7a and, when bound next to the TATA box, hinders transcriptional initiation of *Or98a*. To address this possibility, the Or98a Xbp1 motif was mutated, which produced misexpression across the central antenna (Figure 6D). These data suggest that the differential activity of Xbp1 can be defined by the location of the binding motif in the regulatory regions of the two OR genes.

### The Identified TFs Both Activate and Repress OR Expression

**Figure 7:**
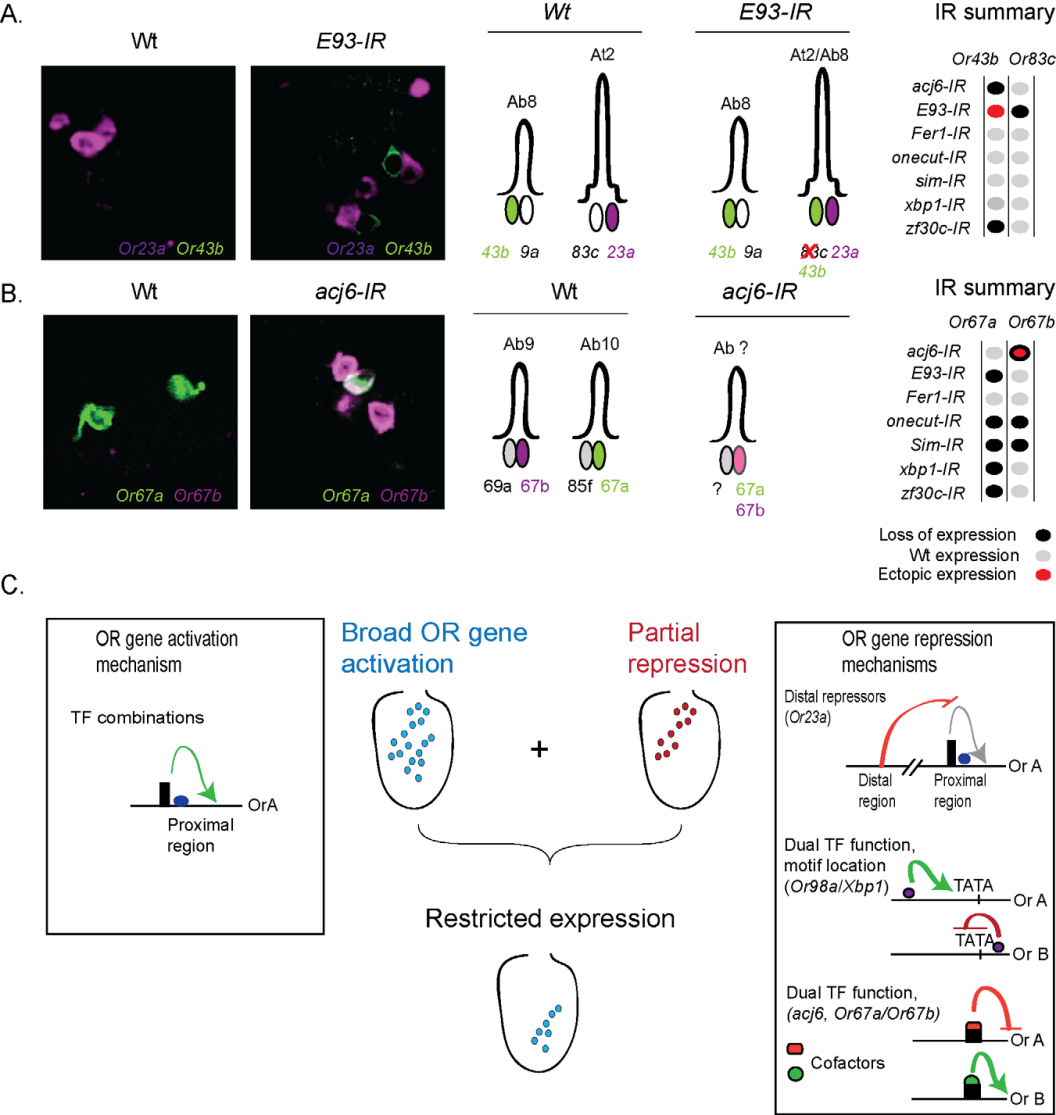
Transcriptional activation and repression are required for correct expression of each OR gene to one OSN class. (A) Double in situ labeling of *Or23a* and *Or43b* in wild type (Wt) and *E93-IR* antennae, the *Or43ba* expression phenotypes are further depicted schematically and summarized as a matrix (grey, wild-type expression; red, ectopic; and black, loss of expression). (B) Double in situ hybridization labeling of *Or67a* and *Or67b* expression in wild type (Wt) and *acj6-IR* antenna. The resultant phenotypes are further summarized as a schematic and a matrix summary. Note the new pair of *Or43b* and *Or23a* when *E93* is knocked down (A), and OR coexpression generated in *acj6* knock-downs (B). (C) Model depicting how activation and repression of OR expression can specify an OSN class. Activation of OR gene expression (left box); different combinations of a limited set of TFs bind a proximal upstream region and produce OR expression in a broad antenna region. Repression of OR gene expression (right box), distal located repressors together with the dual function of the TFs determined by binding site location or possibly cofactor use, restrict OR expression. The combined sum of OR gene activation and repression produce expression to one single OSN class.

To investigate if any of the other six TFs also repress OR gene expression, the knock-downs were reexamined more closely and striking de-repression was observed for two more TFs (Figure 4C). Strong ectopic *Or43b* expression was found in *E93-IR* distal antennae (Figure 7A). Double-labeling experiments showed that OSNs with ectopic *Or43b* expression formed a pair with *Or23a* OSNs and thereby replacing *Or83c* in *E93* knock-downs (Figure 7A), which suggested that *E93* repressed *Or43b* in these OSNs and was required for *Or83c* expression. These results indicate a dual regulatory function similar to Xbp1 in which the location of the unknown E93 motif might possibly produce Or83c expression and Or43b repression. The second example of ectopic expression was identified in *acj6-IR* antennae with *Or67a* being de-repressed and coexpressed with *Or67b* (Figure 7B). Both *Or67a* and *Or67b* have upstream Acj6 binding motifs (Figure 5A) and the TFs required for *Or67b* expression were some of the TFs also required for *Or67a* expression (Figure 7B). Hence, the dual Acj6 function required to separate *Or67a* and *Or67b* expression might be determined in a combinatorial fashion possibly by attraction of different cofactors to each promoter.

## Discussion

We performed a multilevel systematic analysis of sensory class specification in the *Drosophila* olfactory system and identified seven TFs to be critical regulators of odorant gene expression. Different combinations of these TFs are required for precise neuron-specific onset of OR gene expressions as well as maintenance in mature OSNs. The systematic analysis further reveals that the identified TFs bind to different DNA motifs through which they can act as both activators and repressors of OR gene expression (Figure 7C).

### The Seven Identified TFs Are OR Selector Genes

In 1975, Antonio Garcia-Bellido presented the concept of selector genes, TFs that can determine a particular cell fate. Several levels of selector genes has been found, which control gene programs that individually specify organ, tissue, and cell type [27]. Recently, studies in *Caenorhabditis elegans* have revealed that one factor and its motif can be enough to assign expression to one neuronal class [28]–[30]. These observations have led to the formulation of the terminal selector gene hypothesis [31], which put forward that only a small set of TFs are continuously required to express the genes that signify each neuron class, like ORs. However to date few such cases have been identified.

Our systematic analysis presents several observations that suggest the identified TFs to be terminal selector genes for OR expression. First, continuous expression of all seven TFs are required for OR expression in the mature OSNs (Figure 3C). Second, the seven TFs are expressed in the mature OSNs, in various patterns across the antenna (Figure 2B). Third, all 32 tested ORs require different combinations of the TFs for expression (Figure 4A). Last, motifs for the TFs are found upstream of the ORs genes they regulate (Figure 5A) and the motifs are necessary for OR promoter function (Figure 5B). Consequently, it appears that the large number of OR expression patterns are achieved by combinatorial use of a few TFs that function as OR selector genes.

The OR selector genes belong to different protein families (Figure 2A), which indicate that evolution has favored recruitment of TFs with very different DNA binding properties, rather than expansion of one family that shares the basic DNA binding motif, which could secure the fidelity of the combinatorial pattern. On the other hand, the high motif specificity for each factor suggests that loss or gain of motifs for one OR selector might generate a new OR expression pattern and a totally new OSN class. Thus, single OSN class expression and high evolvability, two hallmarks of olfactory system evolution, might in part be due to the combinatorial function of the OR selector genes.

### Single OR Expression, a Large Regulatory Cost

How many OR selector genes are required to uniquely express one OR in each OSN class? We identified seven OR selector genes, but given the limitations of RNAi, it is likely that there are a total of at least ten critical TFs to specify all OSN classes. Even this probably low estimate generates a rather high number of TFs considering that *Drosophila* antennae have 34 OSN classes that express ORs [13]. Theoretically the number of TFs needed for a binary combinatorial code to generate 34 unique outcomes is six (2^6^ = 64). Seven TFs can in theory separate 2^7^ = 128 combinations, and ten TFs designate more than 1,000 combinations, suggesting a large number of unused combinations. This surplus of combinations may be due to the inherent randomness of evolution and the impossibility of creating a streamlined code by chance. Another possibility for this large number is the need for a high degree of fidelity, with little or no ectopic OR expression tolerable for proper functioning of the olfactory system. Extrapolation of our observations to the regulatory requirements of the mammalian olfactory system indicates that at least 200–300 TFs would be required to provide a regulatory system that controls >1,000 mammalian ORs, a daunting number. Therefore, it is reasonable to suspect that the stochastic OR selection mechanism found in vertebrates was added during evolution to accommodate the heavy increase in regulatory costs resulting from an expanded number of OR genes.

### Combinatorial Activation and Repression Control OR Expression

To date very few TFs have been found to be restricted to small neuronal populations in neuroepithelia or in the developing brain in general [32]. This situation has motivated the suggestion that combinatorial TF regulation defines broad expression patterns of molecules such as neurotransmitters, but is insufficient to generate the large number of neuron classes in, for example, the olfactory system [33]. Similarly, all seven selector genes in this study are expressed across the antenna but still are required for the expression of some few ORs (Figures 2B and 4A). How can widely expressed TFs then produce restricted expression patterns? We have formulated two explanations. First, our promoter analysis suggests that the OSN class specificity is in part due to repression. Most ORs have a proximal regulatory region next to the gene that is sufficient for expression in OSNs but requires repression from more distal regions for the spatial restriction to each OSN class (Figure 5D). In this model, the expression of the TFs that produce OR expression does not need to be particularly specific as long as they are counteracted by repressive factors. Second, the identified TFs can both activate and repress OR expression dependent on the location of the binding site or by the available cofactors (Figures 6C, 6D, 7A, and 7B). Dual use of the TFs might increase their regulatory power and as a likely consequence the number of TFs required for OR expression to be reduced. We therefore suggest that specification of large numbers of neuron classes in the olfactory system and likely in the nervous system, require two layers of combinatorial coding, one layer of terminal selector genes that produce expression and a layer of repressors that restrict the expression to each class.

## Materials and Methods

### RNAi Methodology

Virgin flies containing *Pebbled-GAL4*, *UAS-Dicer2*, and the OR promoter fusions were mated with males obtained from the VDRC library. The crosses were set up at 25°C, and after 3 d the parental flies were removed and the vials shifted to 27°C. 2–3 d after eclosure, the GFP levels corresponding to OR expression were ranked 0–5, where 5 corresponded to the wild-type level. For all assays, for five females per line crosses were scored blind to the genotype and all lines with phenotypes scored below 2 were retested. A line was considered to have established phenotype if three consecutive crosses included flies that scored below 2. To further validate the established phenotypes, RNAi lines from the VDRC, NIG, and the Transgenic RNAi Project (TRiP) were used, all the different lines gave the same phenotype as in the screen (Table S1). In order to avoid animals with low RNAi efficiency and reduce the risk of false negatives in the regulatory matrix, OR expression phenotypes were only scored from antennae with total loss of *Or92a* or *Or47b* GFP.

### Mutant Analysis and MARCM

To confirm *acj6* function in OR gene regulation, viable offspring from the *acj6^6^* mutant crossed to the Or92a promoter fusion were analyzed. For the other mutants, genetic mosaics were generated using the MARCM system [34], which was visualized with an Or92a promoter fusion with Gal4 driving the expression of *UAS-SytGFP* [35]. For large clones in the antenna, an *ey-FLP* insertion on the X chromosome was used [36], dependent on gene location mosaics were generated in animals of the following genotypes: *ey-FLP*; *FRT40/42 TF mutant*/*FRT40/42,TubGal80*; *Or92a-Gal4*, *UAS-SytGFP*, or *ey-FLP*; *Or92a-Gal4*, *UAS-GFP*; *FRT80/82 TF mutant*/*FRT80/82 TubGal80*.

### Immunostaining and In Situ Hybridization

Immunostaining and in situ hybridization were performed according to previously described methods [13]; for practical in situ details see [37]. The OR probes were previously used in the OR expression characterization [13]. TF in situ probe templates included sequence from the first coding exon and 1 kb downstream or to the end of the gene and were from genomic DNA and cloned into pBSK. *Or49a*, *Or65a,b,c*, and *Or69a,b* were at the detection limit and excluded from the regulatory matrix analysis.

The primary antibodies used were Rat anti-Elav (7E8A10, DSHB, 1∶500), mouse anti-Acj6 (DSHB, 1∶100), mouse anti-Neuroglian (BP104, DSHB, 1∶50), and Rabbit anti-GFP (TP-401, Torrey Pines, 1∶2,000).

### Bioinformatics

1 kb upstream the translational start site of each OR was scanned with the motifs for HNF6 and BRN3 using weight matrices and programs provided by Genomatix (HNF6.01, BRN3.01, BRN3.02; http://www.genomatix.de/) [38] and Biobase (HNF6_Q6; http://www.gene-regulation.com/) [39]. The Genomatix Matinspector and the Biobase match program optimized matrix thresholds were applied. Putative Xbp1 binding sites were identified on the basis of a pattern search with the consensus motif C/TCACGT [25].

### Electrophoretic Mobility Shift Assay

The various TF DNA binding domains were cloned into the pGEX-2T vector and bacterial recombinant glutathione S-transferase fusion proteins were purified by glutathione Sepharose 4B beads (Amersham). For the binding assay, single-stranded DNA oligonucleotides were end-labeled with T4 polynucleotide kinase (Roche) and G-32-P ATP (PerkinElmer) with T4 polynucleotide kinase according to the manufacturers' instructions, annealed with the complementary strand, and purified on a microspin column (Roche).

Binding reactions were performed at room temperature for 20 min. The binding reaction included 3 µl recombinant glutathione S-transferase fusion proteins, 3 fmol labeled probe, 10 mM HEPES (pH 7.9), 70 mM KCl, 1 mM DTT, 1 mM EDTA, 2.5 mM MgCl2, 4% glycerol, and 1 µg poly (dI/dC) (VWR). Cold competition was performed by adding DNA oligonucleotides in molar excess 15 min before addition of labeled probe. The samples were separated on a 6% acrylamide TBE gel at 60 V for 90 min. Gels were dried and visualized by the FLA-5100 Multi Gauge system (FujiFilm).

### Fly stocks

OR promoter fusion lines have previously been described [13]. *Pebbled*-GAL4 and *acj6^6^*mutant flies were kind gifts from L. Luo. *sim^H9^* was kindly provided by C. Klaembt. *UAS-Dicer2* and the TF-IRs for the screen were provided by the VDRC. Additional TF-IR lines were obtained from NIG and TRiP. *sim-lacZ* flies were obtained from the Szeged *Drosophila* Stock Centre (Szeged, Hungary), and *xbp1^k13803^*, *zf30c^k02506^*, *zf30c-lacZ*, *tubP-Gal80^ts^* were obtained from the *Drosophila* Stock Center (Bloomington, Indiana). For the promoter studies all DNA constructs were injected into *w^1118^* flies, and six to 12 lines were analyzed.

## Supporting Information

Figure S1 **TF knockdown correspond to loss of TF mRNA.** In situ labeling of each TF (red) and DAPI (blue) performed on TF-IR antenna, note the tight correlation of loss of *xbp1* (red) and *Or92a-CD8::GFP* expression (green).
(TIF)

Figure S2 **Olfactory sensory neuron layers and morphology are unperturbed in the TF knock downs.** Antenna from TF-IR flies stained for neuronal markers in red (Neuroglian and Elav) and counterstained with DAPI.
(TIF)

Figure S3 **None of the seven TFs were required for OR expression to one antenna domain or sensilla group.** Regulatory matrix arranged after the five antenna domains (blue to red) and each sensilla group. Each domain is exemplified by one OR promoter fusion in green, counterstained with ELAV in red. Note that at least three of the seven TFs are required for expression in each sensilla group (basiconic, trichoid, and coeloconic).
(TIF)

Figure S4 **Predicted DNA motifs are bound by the identified TFs in vitro.** (A) Electrophoretic mobility shift assay (EMSA) performed with radiolabeled probe containing the vertebrate Xbp1 core sequence with (+) or without (−) recombinant Xbp1. Increasing amounts (100-, 200-, 300-, 900-fold excess) of nonlabeled probe were used as cold competitors; 900-fold excess of a probe carrying a mutated motif is shown in the lane labeled “m.” (B) Acj6, onecut, and Xbp1 motifs upstream of four ORs. (C) Expanded EMSA validation of the predicted Acj6, Xbp1, and onecut motifs from the four OR promoters. Radiolabeled motif probe (P) and competition with cold (C) motif probe at 900-fold excess are shown.
(TIF)

Table S1 **All tested IRs and mutants for each TF gave rise to identical phenotypes.** Statistics related to Figure 1. OR expression phenotypes for two or more TF-IRs and available mutants for each gene, noted as number of animals with loss of OR expression/number of analyzed animals. Wt, wild type, denotes no loss of expression.
(DOC)

Table S2 **TF-IRs phenotypes for the 32 ORs in the regulatory matrix.** Statistics related to Figure 3. OR expression detected by in situ hybridizations on TF-IRs antennas and rated per animal from; 0 (loss) to 5 (control levels) and denoted as phenotype level/animals. OR expression rated above 2 was considered to be wild-type variance.
(XLS)

Table S3 **Motifs upstream all 32 analyzed ORs.** Statistics related to Figure 5A. Motif location is denoted as bps upstream the translation start for each OR gene and motifs found downstream the TATA box are depicted with an asterisk.
(DOC)

## Abbreviations

IR: inverted repeat
OR: odorant receptor
OSN: olfactory sensory neuron
