# Supplementary figures and images for: Combinatorial Activation and Repression by Seven Transcription Factors Specify Drosophila Odorant Receptor Expression
Source: PLoS Biol. 2012 Mar 13;10(3):e1001280. doi: 10.1371/journal.pbio.1001280 (PMC3302810; doi:10.1371/journal.pbio.1001280)

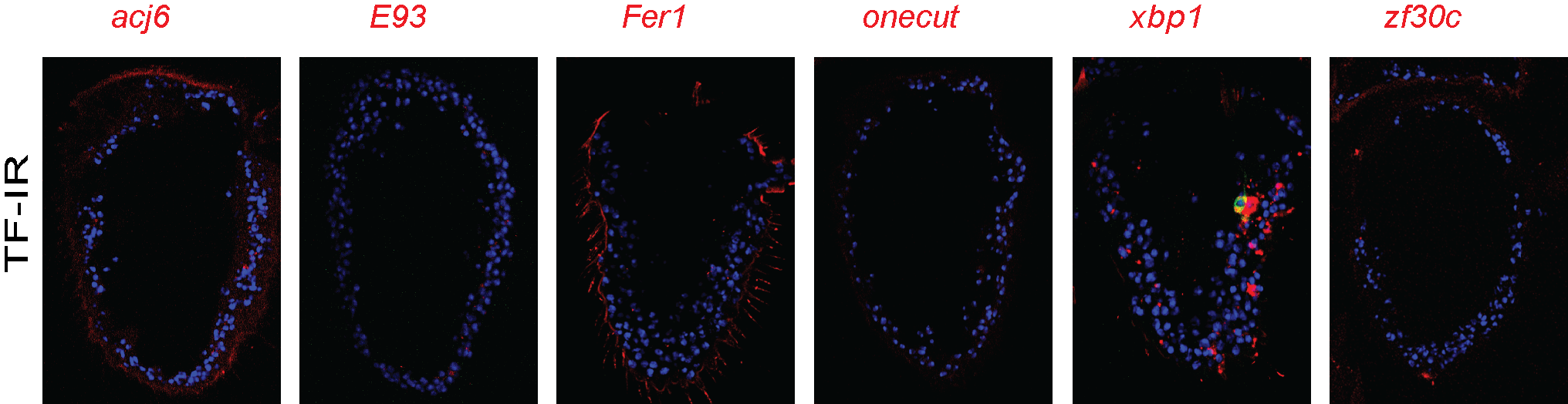

Supplement: Figure S1 — TF knockdown correspond to loss of TF mRNA. In situ labeling of each TF (red) and DAPI (blue) performed on TF-IR antenna, note the tight correlation of loss of xbp1 (red) and Or92a-CD8::GFP expression (green). (TIF) [file pbio.1001280.s001.tif]

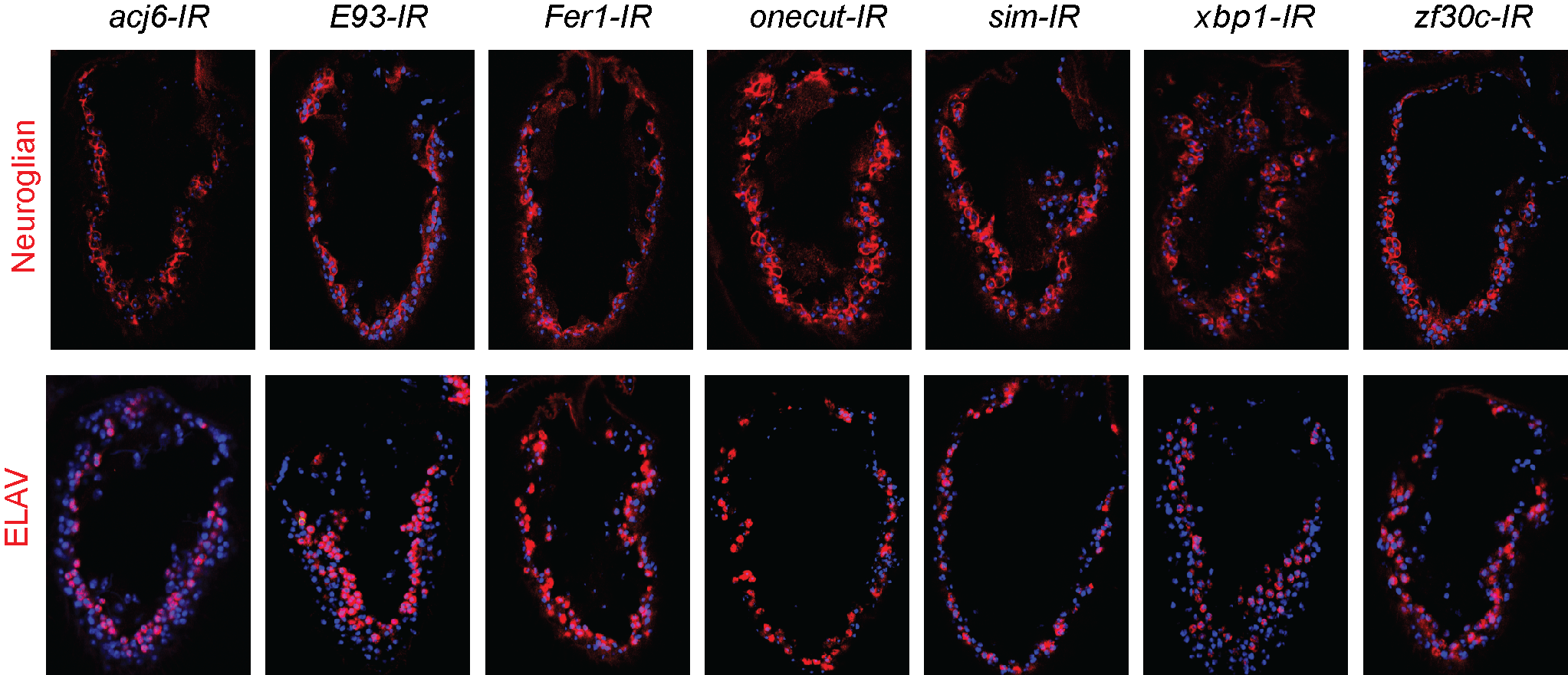

Supplement: Figure S2 — Olfactory sensory neuron layers and morphology are unperturbed in the TF knock downs. Antenna from TF-IR flies stained for neuronal markers in red (Neuroglian and Elav) and counterstained with DAPI. (TIF) [file pbio.1001280.s002.tif]

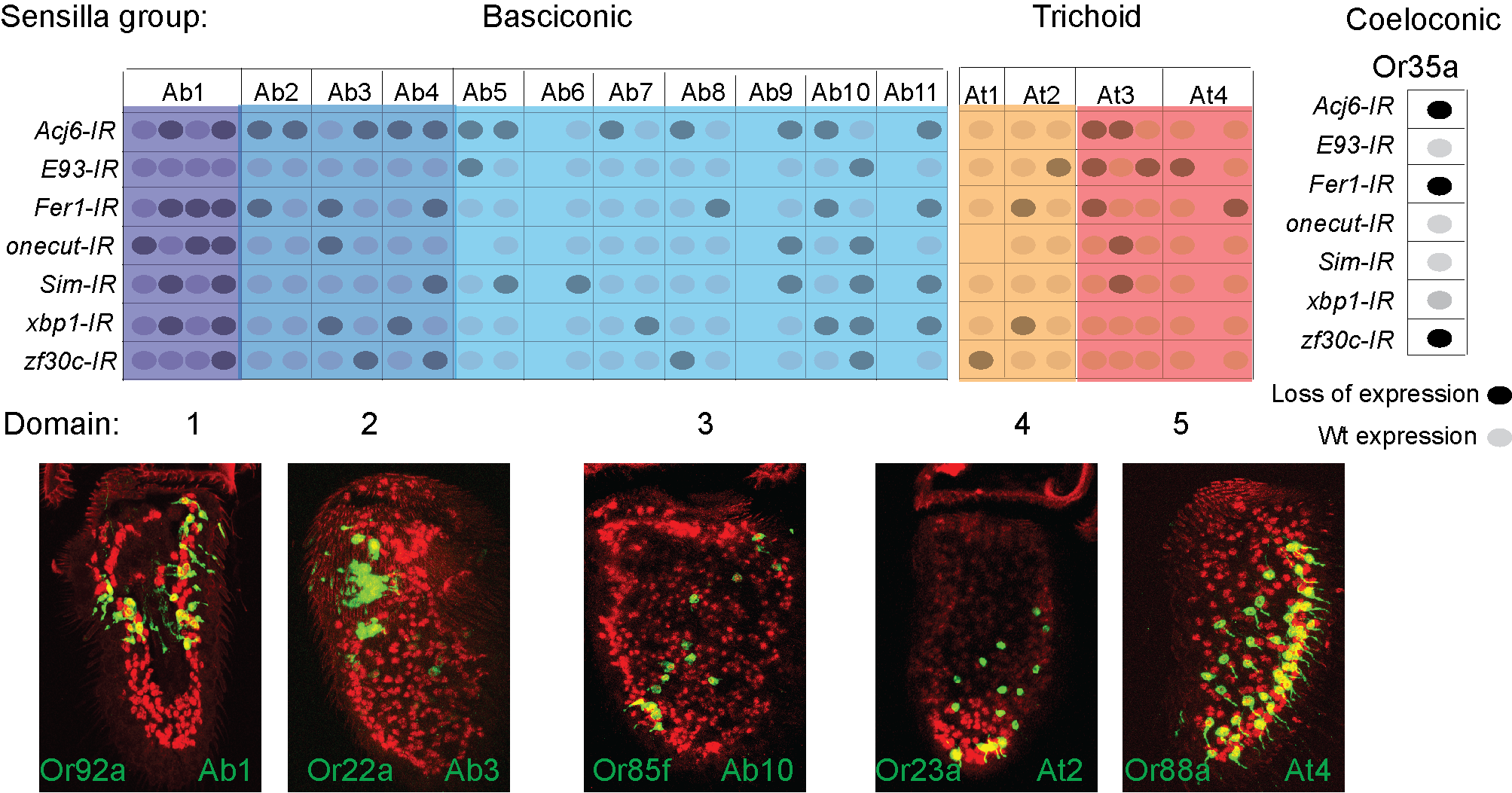

Supplement: Figure S3 — None of the seven TFs were required for OR expression to one antenna domain or sensilla group. Regulatory matrix arranged after the five antenna domains (blue to red) and each sensilla group. Each domain is exemplified by one OR promoter fusion in green, counterstained with ELAV in red. Note that at least three of the seven TFs are required for expression in each sensilla group (basiconic, trichoid, and coeloconic). (TIF) [file pbio.1001280.s003.tif]

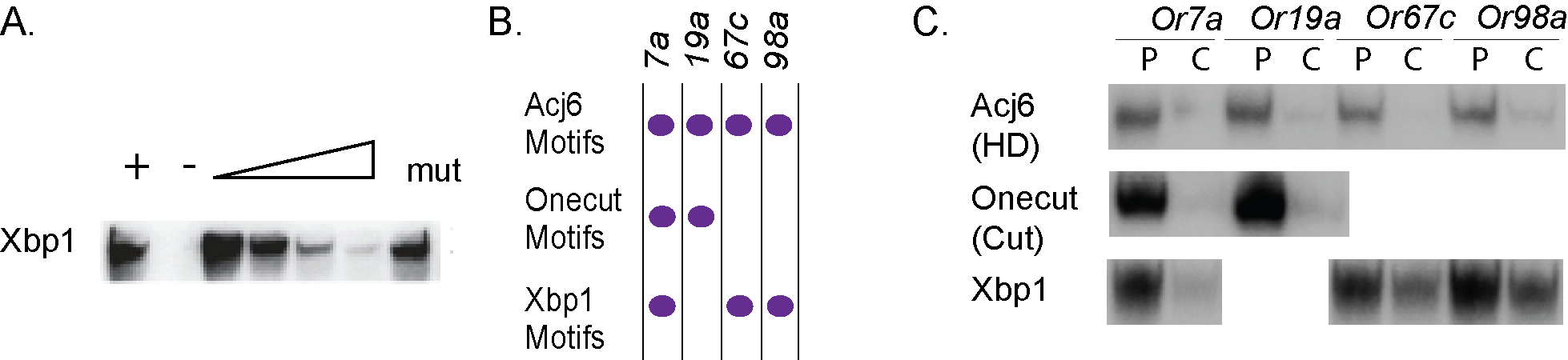

Supplement: Figure S4 — Predicted DNA motifs are bound by the identified TFs in vitro. (A) Electrophoretic mobility shift assay (EMSA) performed with radiolabeled probe containing the vertebrate Xbp1 core sequence with (+) or without (−) recombinant Xbp1. Increasing amounts (100-, 200-, 300-, 900-fold excess) of nonlabeled probe were used as cold competitors; 900-fold excess of a probe carrying a mutated motif is shown in the lane labeled “m.” (B) Acj6, onecut, and Xbp1 motifs upstream of four ORs. (C) Expanded EMSA validation of the predicted Acj6, Xbp1, and onecut motifs from the four OR promoters. Radiolabeled motif probe (P) and competition with cold (C) motif probe at 900-fold excess are shown. (TIF) [file pbio.1001280.s004.tif]
