# Supplementary material for: Combinatorial Activation and Repression by Seven Transcription Factors Specify Drosophila Odorant Receptor Expression
Source: PLoS Biol. 2012 Mar 13;10(3):e1001280. doi: 10.1371/journal.pbio.1001280 (PMC3302810; doi:10.1371/journal.pbio.1001280)
Supplement: Table S1 — All tested IRs and mutants for each TF gave rise to identical phenotypes. Statistics related to Figure 1. OR expression phenotypes for two or more TF-IRs and available mutants for each gene, noted as number of animals with loss of OR expression/number of analyzed animals. Wt, wild type, denotes no loss of expression. (DOC) [file pbio.1001280.s005.doc]

|  | VDRC | | | NIG-Fly | | | TriP | | | Mutants | |
| --- | --- | --- | --- | --- | --- | --- | --- | --- | --- | --- | --- |
|  | line | OSN class | | line | OSN class | | line | OSN class | | line | OSN class |
|  |  | Or92a | Or47b |  | Or92a | Or47b | Bloomington# | Or92a | Or47b |  | Or92a (VA2) |
| *acj6* | 49A10 | loss | Wt | Not available |  |  | 29335 | 4/16 | Wt | *Acj6^6^* | 20/20 |
| *E93* | 47A5 | Wt | loss | 18389R-1 | Wt | 3/27 | Not available |  |  | Not available |  |
| *Fer1* | 140D4 | loss | Wt | 10066R-4 | 5/8 | Wt | 27737 | 8/18 | Wt | Not available |  |
| *onecut* | 47C8 | loss | Wt | 1922R-2 | 2/10 | Wt | 29343 | 7/12 | Wt | Not available |  |
| *sim* | 138C6 | loss | Wt | Not available |  |  | 26739 | 5/14 | Wt | *sim^H9^* | 14/14 |
| *xbp1* | 50A6 | loss | Wt | Not available |  |  | 25990 | 9/15 | Wt | *xbp1^k13803^* | 2/25 |
| *zf30c* | 46D10 | loss | Wt | 3998R-1 | 3/18 | Wt | 35678 | 2/12 | Wt | *zf30c^k02506^* | 9/23 |
