# Supplementary material for: Combinatorial Activation and Repression by Seven Transcription Factors Specify Drosophila Odorant Receptor Expression
Source: PLoS Biol. 2012 Mar 13;10(3):e1001280. doi: 10.1371/journal.pbio.1001280 (PMC3302810; doi:10.1371/journal.pbio.1001280)
Supplement: Table S3 — Motifs upstream all 32 analyzed ORs. Statistics related to Figure 5A. Motif location is denoted as bps upstream the translation start for each OR gene and motifs found downstream the TATA box are depicted with an asterisk. (DOC) [file pbio.1001280.s007.doc]

|  | Acj6 | Onecut | Xbp1 |
| --- | --- | --- | --- |
| ORs | Motif location | Motif location | Motif location |
| 2a | -221 | -15* |  |
| 7a | -411 | -33* | -947 |
| 9a |  | -228 |  |
| 10a |  | -113, -282 |  |
| 13a | -401 |  |  |
| 19a | -216 | -153 |  |
| 22a | -435 | -379, -496, -767 | -595, -728 |
| 23a |  |  | -656 |
| 35a |  |  |  |
| 42b | -136 |  | -871 |
| 43a |  |  |  |
| 43b | -85, -527 |  |  |
| 47a | -403 |  |  |
| 47b |  |  |  |
| 49b |  | -231, -441 |  |
| 56a | -219 | -78 |  |
| 59b | -213 |  |  |
| 67a | -305 | -765 |  |
| 67b | -159 | -281, -446 |  |
| 67c | -184 |  | -716 |
| 67d | -39*, -148 |  | -554 |
| 82a | -97, -177 | -74 |  |
| 83c |  |  |  |
| 85a | -96, -242 | -18* |  |
| 85b | -698 |  |  |
| 85f | -386 |  | -329 |
| 88a |  |  |  |
| 92a | -95, -677 | -154 | -967 |
| 98a | -292 | -108 | -52* |
| Gr21a |  | -204 |  |

*Motif downstream TATA box
